# Effect of Showing Angiograms to Patients After Elective Percutaneous Coronary Intervention on Anxiety and Illness Perception: A Randomized, Blinded, Controlled Clinical Trial

**DOI:** 10.31661/gmj.v8i0.1556

**Published:** 2019-10-26

**Authors:** Babak Geraiely, Roya Sattarzadeh Badkoubeh, Maryam Jalalsafari, Nazila Shahmansouri, Anahita Tavousi, Nima Nazari, Seyedeh Hamideh Mortazavi

**Affiliations:** ^1^Tehran Heart Center, Tehran University of Medical Sciences, Tehran, Iran; ^2^Department of Cardiology, Imam Khomeini Hospital, Tehran University of Medical Sciences, Tehran, Iran; ^3^Department of Psychiatry, Tehran Heart Center, Tehran University of Medical Sciences, Tehran, Iran; ^4^Department of Anesthesiology, Imam Khomeini Hospital, Tehran University of Medical Sciences, Tehran, Iran

**Keywords:** Coronary Heart Disease, Percutaneous Coronary Interventions, Anxiety, Questionnaires

## Abstract

**Background:**

As an invasive modality, a coronary angioplasty may cause a great deal of anxiety in patients and affect their mental health and general well-being. Accordingly, we sought to assess whether showing patients the video of their elective percutaneous coronary intervention (angiogram) could affect their illness perception and anxiety level.

**Materials and Methods:**

In this randomized clinical trial, the patients undergoing angioplasty, were randomly divided into two groups of 30 patients. Angiograms were shown only to the intervention group postprocedurally. A checklist comprising demographic data and clinical presentations as well as the Beck anxiety questionnaire and the Brief Illness Perception Questionnaire (BIPQ) was completed for each patient immediately after the intervention and one month later. The differences in the patients’ anxiety level and illness perception were analyzed.

**Results:**

In the intervention group, the mean anxiety score before and after watching the angiograms was 34.26 ± 8.1 and 24.4 ± 8.56, respectively. While in the control group, the score before and after angioplasty was 34.46 ± 9.34 and 26.6 ± 9.44, respectively. Thus, watching angiograms led to a significant decrease in the anxiety score in the intervention group, whereas there was no such difference in the control group. There was also a considerable difference in the anxiety score between the two groups. Further, there was a significant decrease in the BIPQ score of the intervention group after watching the angioplasty videos.

**Conclusion:**

Educating cardiovascular patients about diagnostic and therapeutic procedures may confer such good outcomes as alleviated anxiety, enhanced satisfaction, and ultimately, fewer anxiety-related complications.

## Introduction

Nowadays, cardiovascular disease is one of the most common health problems and the leading cause of death around the globe [1-3]. Various revascularization techniques are currently in wide use for the treatment of patients with coronary artery disease. Similar to many treatment procedures, percutaneous coronary intervention (PCI) is associated with some complications such as myocardial infarction, stroke, transient ischemic attack, renal failure, and even death [4-6] and thus patient anxiety and discomfort. Anxiety is the most common mental disorder and is a prevalent psychological reaction in response to new changes and experiments. Depression and anxiety are common in patients with cardiovascular disease. Research has indicated that anxiety, along with certain other condition, can exacerbate cardiovascular disease and should be deemed a strong and independent risk factor for mortality in cardiovascular disease [7, 8]. Cardiovascular patients undergoing the invasive modality of angioplasty are prone to experience anxiety, which could affect both their psychological state and their underlying cardiovascular disease. We hypothesized that a comprehensive explanation of the angioplasty procedure to patients—including showing an angioplasty procedure video—might alleviate their level of anxiety and reduce their signs and complications, leading to not only a decrease in unnecessary visits and medical expenses but also an improvement in satisfaction and quality of life. We, therefore, endeavored to assess whether showing patients the angioplasty procedure movie (angiogram) of their elective PCI could positively affect their anxiety level and illness perception.

## Materials and Methods

### Patients

**Figure 1:**
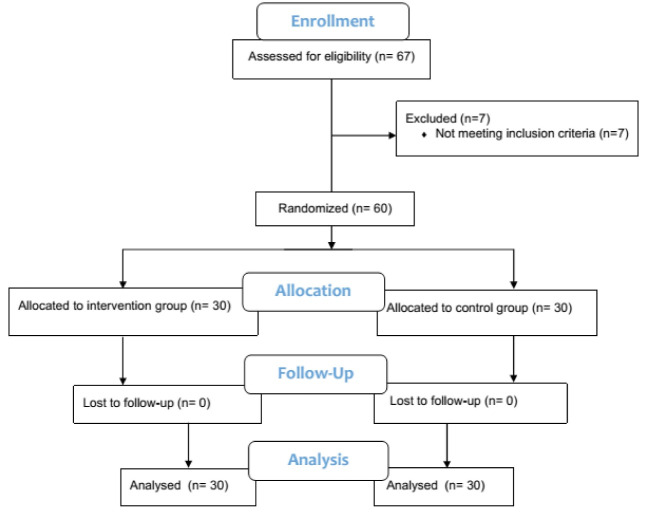
Study flow diagram

The present randomized clinical trial recruited patients aged between 40 and 65 years candidate for elective PCI following coronary angiography at Imam Khomeini Hospital, Tehran, Iran (Figure-1). Patients were excluded if they had psychological disorders necessitating pharmacological treatments, history of hospitalization, hypo- or hyperthyroidism, postprocedural complications, age below 40 years or above 65 years, illiteracy, history of coronary angiography, heart failure, ejection fraction below 30%, and candidacy for medical therapy. Those who met the inclusion criteria were randomly assigned to the intervention group (n=30) and the control group (n=30). The sampling method was a simple selection.

### Questionnaires

The Beck anxiety inventory was evaluated in the Iranian population by [9] who reported a good reliability (r=0.72, P<0.001), a very good validity (r=0.83, P<0.001), and an excellent internal consistency (α=0.92). This questionnaire consists of 21 questions (4 selection switches for each question); consequently, the score range is between 0 and 63. Scores from 8 to 15 represent low anxiety levels, from 16 to 26 intermediate anxiety levels, and from 26 to 63 high anxiety levels. The reliability and validity of the Persian version of the BIPQ were evaluated by Bazzazian and Besharat [10]. The BIPQ has eight items, with all the items—except the causal question—rated using a scale of 0 to 10. Five of the items assess cognitive illness representations: consequence (Item 1), timeline (Item 2), personal control (Item 3), treatment control (Item 4), and identity (Item 5). Two of the items assess emotional representations: concern (Item 6) and emotions (Item 8). One item investigates illness comprehensibility (Item 7). The scores of questions 3, 4, and 7 were reversed and added to the scores of the other questions. Higher scores denote worse disease perceptions.

### Data Collection

A checklist comprising demographic data, place of residence, level of education, comorbidities, family history, and the initial complaint was filled at admission. Preprocedural, the procedure, nature of the disease, and the involved vessels were explained to the patients in the intervention group, while the control group received information only about the number of involved vessels and angioplasty success rates. Postprocedural, only the patients in the intervention group were shown the video of their own angioplasty (angiogram). All of the abovementioned tasks were performed by a cardiology resident. The patients in both groups completed the Beck anxiety inventory (21 questions) and the Brief Illness Perception Questionnaire (BIPQ; 8 questions). At one month’s follow-up, another checklist was completed for all the patients once again if they met none of the exclusion criteria such as having infection, hematoma, and pseudoaneurysm of the puncture site. The checklist contained information on the number of chest pain and palpitation episodes within the preceding month, Beck anxiety inventory, and the BIPQ. After that, illness perception and anxiety levels at one month’s follow-up were compared between the intervention and control groups. Also, the operators were blinded to the study groups.

### Ethical Issues

The research protocol was approved by the Institutional Review Board and the Ethics Committee of Imam Khomeini Hospital. Informed consent was obtained from the entire study population, and the study protocol conforms to the ethical guidelines of the 1975 Declaration of Helsinki. The trial was registered in the Iranian Registry of Clinical Trials (http://irct.ir) with an IRCT registration number of IRCT2017021932666N1 and ethical code of IR.TUMS.IKHC.REC.1395.1669.

### Statistical Analysis

All the analyses were performed using the IBM SPSS Statistics for Windows, version 19.0 (Armonk, NY: IBM Corp). The data were compared between the two groups using the chi-square test and the McNemar test. A P-value of less than 0.05 was considered statistically significant.

## Results

The present study evaluated 60 patients who the candidate for elective PCI following coronary angiography. The demographic data of the patients are depicted in Table-1. As demonstrated, the two groups were similar in baseline characteristics. As is depicted in Table-2, the mean Beck anxiety score was 34.26 ± 8.1 in the intervention group and 34.46 ± 9.34 in the control group at baseline while it was 24.4 ± 8.56 in the intervention group after watching the video of the procedure and 26.6 ± 9.44 in the control group. The difference in the mean Beck anxiety score was statistically significant between the two groups (P=0.033). The BIPQ score was 60.63 ± 8.43 in the intervention group and 57.83 ± 10.72 in the control group at baseline and 47.16 ± 8.29 in the intervention group after watching the video of their angiograms and 56.33 ± 8.05 in the control group (P=0.0001).

## Discussion

In the present study, we concluded that watching angiograms could lead to a significant decrease in the anxiety score in the intervention group, whereas there was no such difference in the control group. Furthermore, there was a significant decrease in the BIPQ score of the intervention group after watching their angioplasty videos. A growing number of patients with cardiovascular disease are currently followed up around the world by advanced diagnostic and therapeutic methods. Increasing rates of urban life, obesity, smoking, and occupational and psychosocial stress combined with decreased physical activity and inattention to health advice have increased cardiovascular disease burden and mortality. What further compounds the matter is the negative impact of the disease on quality of life in cardiovascular patients. Indeed, since cardiovascular disease is a chronic state associated with high health care costs, it is beneficial to focus on improving patients’ quality of life [11]. PCI is the most common treatment modality in coronary revascularization. In the current study, we sought to determine whether showing patients their angiograms would reduce their level of anxiety. The main goal of our study was to assess the patients’ anxiety levels after watching a video of their angioplasty procedure. One of the main sources of anxiety is hospitalization, and the feeling escalates when the patient is a candidate for an invasive procedure such as PCI. Anxiety is an emotional status accompanied by tension, anger, fear, and activated autonomic system—leading to psychophysical responses [12]. Anxiety increases psychological, and then physiological activities such as heart rate, respiratory rate, blood pressure, and cardiac output, and these changes can be harmful to patients with cardiovascular disease. Excess anxiety can also delay the recovery period [13]. The results of the present study demonstrated no significant difference in the baseline demographic and clinical characteristics between the intervention and control groups. We found a significant difference in the Beck anxiety score between two study groups at one month’s follow-up, although the difference was more prominent in the intervention group. One possible explanation for this finding is that the reduced Beck anxiety score in the intervention and control group may have been in consequence of the passage of the recovery period and improvement in the patients’ quality of life after coronary angioplasty. The study of Suprakash *et al*. [14], assessing the relationship between depression, anxiety, and quality of life and outcomes after percutaneous transluminal coronary angioplasty (PTCA), showed that 46% of their patients had significant anxiety and 32.1% had significant depression before PTCA. Following successful PTCA, none of the patients had significant anxiety, and only 2 (3.6%) had significant depression. Farkhondeh *et al.*[15], investigated the effects of discharge planning on stress, anxiety, and depression in patients who had undergone PTCA. They showed a statistically significant decrease in the patients’ level of stress, anxiety, and depression one month after the planned discharge. Although the scores of stress, anxiety, and depression in the experimental group did not differ significantly on the day of discharge, the decrease was considerable compared with that of the control group. Cengiz *et al.*[16], evaluated the effects of audiovisual education prior to coronary angiography on patients’ anxiety state. The authors provided an audiovisual educational program for their experimental group (60 patients) on the day before the procedure, while they gave their control group (60 patients) only verbal explanations about the procedure. They found that the level of anxiety, pulse rate, systolic and diastolic blood pressures were significantly lower in the experimental group than in the control group. Shiloh *et al.*[17], investigated the effects of instruction during coronary intervention on the emotional, cognitive, and behavioral outcomes of patients undergoing PCI. They provided explanations to their intervention group patients while they were watching the monitor. These patients reported less negative affect and anxiety and higher levels of self-efficacy than the control group at one month’s follow-up. A randomized clinical trial by Ruffinengo *et al.*[18], evaluated the influence of informative video on the level of anxiety in patients who underwent coronary angiography. The anxiety level of the patients, as assessed with the Spielberger scale, was significantly reduced in the experimental group. These patients also reported higher levels of satisfaction than the control group. Chiming in with the results of similar studies, our findings demonstrated that educating patients about the procedure and the therapeutic process alleviated their anxiety and enhanced their quality of life. Nevertheless, the educational method drawn upon in the current study is a novel method by comparison with those used in the previous investigations. We believe that this simple method is also capable of reducing unnecessary visits and costs related to patients’ anxiety. One of the factors assessed in our research was illness perception in the intervention and control groups. Illness perception is a very important component in chronic diseases and may be involved in disease-related functioning and outcomes in adults [10]. The results of our study showed that there was a significant difference in illness perception between the intervention and control groups based on the BIPQ. As was explained previously, higher scores of BIPQ indicate patients’ poorer perception of their illness. It is worthy of note that illness perception in our control group also decreased, but the change failed to constitute statistical significance. One possible explanation for this is that these patients may have received education from their nurses and treating physicians during their hospital stay. To the best of our knowledge, there is no similar study in the existing literature on illness perception in patients undergoing coronary angioplasty, and the present work is the first study in this field. The results of previous studies have indicated that psychological problems, not least depression and anxiety, have a prominent role in the outcome of cardiovascular patients. Similar to depression, anxiety is one of the most common psychological reactions in patients with cardiovascular disease and heart failure [19]. Left untreated, anxiety can increase the risk of cardiac events. It is, therefore, advisable that strategies be devised to lessen anxiety levels and as such prevent complications in this group of patients.

## Study Limitations

Small sample size could be mentioned as the limitation of the current study. Several factors influence the anxiety of the patients, such as smoking and multimorbidity. However, unfortunately, we did not have the data of these variables for all of the patients and could not use them in the final analysis.

## Conclusion

The results of the present study indicated that showing patients a video of their angioplasty procedure alleviated their anxiety level; it can, therefore, be considered a novel inexpensive method for the reduction of further anxiety-related complications. However, further investigations with larger sample sizes and longer-term follow-up periods are needed to shed sufficient light on this issue.

## Conflict of Interest

None.

**Table 1:** Demographic Characteristics of Patients Admitted for Elective Percutaneous Coronary Intervention in Intervention and Control Groups

**Variables**	**Intervention group**	**Control group**	**P-value**
**Age (year)**	56.13±10.12	58.5±7.44	0.307
**Gender (male)**	20 (66.7)	18 (60)	0.78
**Marital status**	
Single	2 (6.7)	1 (3.3)	0.55
Married	28 (93.3)	29 (96.7)	
**Education level (years)**	
>12	16 (53.3)	14 (46.7)	0.67
12-16	12 (40)	15 (50)	
≥16	2 (6.7)	1 (3.3)	
**Residence**	
Urban	29 (96.7)	29 (96.7)	1.0
Rural	1 (3.3)	1 (3.3)	
**Number of chest pain episodes**	11.4±7.82	9.73±6.7	0.379
**Number of palpitation episodes**	3.36±6.28	3.33±4.44	0.408
**Family history**	8 (26.7)	5 (16.7)	0.347
**Underlying disease**	24 (75)	21 (70)	0.37

Data are shown as Mean±SD, or number (%)

**Table 2:** Beck Anxiety Score and the BIPQ Score in the Intervention and Control Groups before and after Showing the Angioplasty Procedure Video to the Intervention Group

	**Variables**	**Intervention group**	**Control group**	**Statistical estimates between the groups**
**Anxiety**	Before showing the video	34.26±8.1	34.46±9.34	t=0.28, P=0.778
	After showing the video	24.4±8.56	26.6±9.44	t=0.889, P=0.474
	Statistical estimates within the groups	t=8.89, P=0.0001	t=8.59, P=0.0001	
	Changes in the scores	-9.86±6.07	-6.86±4.37	t=2.19, P=0.033
**BIPQ**	Before showing the video	60.63±8.43	57.83±10.72	t=1.12, P=0.266
	After showing the video	47.16±8.29	56.33±8.05	t=4.34, P=0.0001
	Statistical estimates within the groups	t=10.88, P=0.0001	t=1.19, P=0.243	
	Changes in the scores	-13.66±6.77	-1.5±6.89	t=6.78, P=0.0001

Data are shown as mean±SD, or number (%) **BIPQ:** Brief Illness Perception Questionnaire
